# Establishing Diagnostic Reference Levels for Paediatric CT Imaging: A Multi-Centre Study

**DOI:** 10.3390/healthcare13141728

**Published:** 2025-07-17

**Authors:** Yassine Bouchareb, Manar Al Kharusi, Amani Al Maqbali, Amal Al Maimani, Hasina Al Maskari, Srinivasa Rao Sirasanagandla, Amna Al Jabri, Faiza Al Kindi, Saud Al Shabibi, Saleh Baawain

**Affiliations:** 1 Department of Radiology and Molecular Imaging, College of Medicine and Health Sciences, Sultan Qaboos University, Muscat 123, Oman; amna1@squ.edu.om; 2 Department of Physics, College of Science, Sultan Qaboos University, Muscat 123, Oman; 3 Department of Radiology and Molecular Imaging, Sultan Qaboos University Hospital, Muscat 123, Omanssab@squ.edu.om (S.B.); 4 Department of Radiology, Royal Hospital, Muscat 123, Oman; 5 Department of Human and Clinical Anatomy, College of Medicine and Health Sciences, Sultan Qaboos University, Muscat 123, Oman; srinivasa@squ.edu.om

**Keywords:** diagnostic reference level, paediatric, computed tomography, CTDI_vol_, DLP

## Abstract

**Background:** Computed Tomography (CT) imaging is widely recognised for its high capability in assessing multiple organs. However, concerns about patient radiation exposure, particularly in children, pose significant challenges. **Objective:** This study aimed to establish diagnostic reference levels (DRLs) for paediatric patients in the most common CT examinations to monitor and better control radiation doses. **Methods:** Dosimetry records from 5956 patients’ scans for the four most common CT imaging examinations—Head, Chest, Abdomen Pelvis (AP), and Chest Abdomen Pelvis (CAP)—were considered. The CT dosimetric quantities (CT dose-index volume (CTDI_vol_) and dose-length product (DLP)), along with patient demographics (age and weight), were collected from radiology data storage systems. DRLs for CTDI_vol_ and DLP were determined for each imaging examination, stratified by patient age and weight groups, in accordance with ICRP recommendations. **Results:** The derived DRLs are presented as [median CTDI_vol_ (mGy): median DLP (mGy·cm)]. For (<1 yr): Head: 13:187, Chest: 0.4:7, AP: 0.9:19, CAP: 0.4:10. For (1–5 yrs): Head: 16:276, Chest: 1:22, AP: 1.5:58, CAP: 1.6:63. For (6–10 yrs): Head: 19:332, Chest: 1.4:35, AP: 1.9:74, CAP: 2:121. For (11–15 yrs): Head: 21:391, Chest: 3:86, AP: 4.1:191, CAP: 3:165. We observed that both the CTDI_vol_ and DLP DRL values increase with patient age. Weight-based DRLs follow similar trends for CTDI_vol_, while DLP values show noticeable variations in Chest and AP examinations. **Conclusions:** The study findings highlight the need for review and optimisation of certain scanning protocols, particularly for chest and AP examinations. The derived DRLs are consistent with findings from other studies. The study recommends establishing national paediatric DRLs to enhance radiology practice across the country and ensure adherence to international safety standards.

## 1. Introduction

In recent decades, Computed Tomography (CT) has become one of the most vital diagnostic imaging techniques in the medical field [1]. It provides fast, non-invasive, and highly detailed images, which are essential for screening, diagnosing, and monitoring various health conditions [2,3]. CT imaging is used extensively for both adults and paediatric patients due to its ability to assess multiple organs and structures, helping doctors make more informed decisions [4]. Currently, the increased utilization of CT scans in both developed and developing countries poses great concerns about the radiation hazards associated with these CT examinations [5]. Despite its tremendous clinical value, the reports have demonstrated that CT examinations can promote cancer risk due to associated ionizing radiation [6,7]. As the utility of the CT has been greatly increasing over the years, the sharp increase in the volume of CT imaging examinations was found to be the major source of radiation exposure in the medical field [1]. A study by UNSCEAR on overall effective doses related to diagnostic medical exposures revealed that CT examinations represent 9.6% of total radiological investigations and contribute to 61.6% of total effective doses [6]. While the benefits of CT scans are undeniable, concerns about patient radiation exposure, especially for children, have raised significant challenges in healthcare practices [8]. Paediatric patients, being more sensitive to ionizing radiation, are at a higher risk of potential radiation-induced harm, necessitating the implementation of strategies that limit exposure while maintaining diagnostic effectiveness [9].

The International Commission on Radiological Protection (ICRP) introduced the concept of Diagnostic Reference Levels (DRLs) as a tool to optimize radiation doses in medical imaging procedures [10,11,12]. DRLs are used globally to ensure that the radiation doses administered to patients are neither excessively high nor low, serving as a benchmark for optimal radiation exposure [13,14]. In the last two decades, paediatric DRLs have been established in various countries for the most common CT examinations, including CT Head, CT Chest, CT Chest Abdomen Pelvis (CAP), and CT Abdomen Pelvis (AP) [15,16,17,18]. DRLs have been proven to be effective tools in monitoring radiation exposure and optimizing patient safety during diagnostic imaging [19].

Several studies have focused on establishing and updating DRLs for paediatric CT imaging. In the UK, a review in 2011 updated national DRLs, showing a reduction in radiation doses over time, with a 17–47% decrease by 2019 due to advances in CT technology [20]. In France and Ireland, studies revealed significant variations in radiation doses, highlighting the need for standardization [21]. The American College of Radiology also updated DRLs in 2021 for the top 10 paediatric CT exams [22,23]. These studies show the importance of regularly updating DRLs to ensure patient safety and diagnostic effectiveness.

The recommended DRL quantities for CT imaging examinations are CT dose-index volume (CTDI_vol_) and dose-length product (DLP). The CTDI_vol_ value represents the radiation dose resulting from a single rotation of the X-ray tube, while DLP indicates the total radiation exposure of the patient, and is highly affected by length of imaged area, called scan length or imaging range [23,24,25,26]. Additionally, regular review of local DRLs for CT imaging examinations is essential to maintain compliance with radiation safety policies, and ensure that DRLs are not systematically exceeded, thereby enhancing radiology practice [25,27]. The fast growth of CT technology, such as spectral CT and high-slice scanners, further reinforces the importance of establishing DRLs, considered as one of the hottest topics in current radiology practice, as an efficient and valuable tool for radiation dose optimization [12,26].

However, while DRLs have been widely established in many regions, specific attention to paediatric CT imaging remains limited. Children are more susceptible to radiation damage due to their growing tissues, smaller size, and longer expected lifespan. Therefore, it is imperative to develop and evaluate DRLs specifically for paediatric patients to ensure that their radiation exposure is minimized without compromising diagnostic quality [11,28]. Existing studies on DRLs for paediatric CT have often been generalized from adult populations or focused on specific diagnostic procedures.

Oman has made significant strides in improving healthcare services, ensuring the safe use of diagnostic imaging for children remains a priority. The Sultan Qaboos University Hospital (SQUH) and Royal Hospital (RH), two of the largest healthcare facilities in Oman, provide a wide range of imaging services, including paediatric CT scans. However, the absence of locally established DRLs for paediatric patients necessitates the development of these reference levels to improve the local radiation protection practice and ensure compliance with international safety standards.

This study aimed to establish DRLs for paediatric CT imaging by evaluating the radiation doses from routine paediatric CT examinations. The main goal was to estimate DRL values for the four most common paediatric CT examinations, including CT Head, CT Chest, CT Abdomen Pelvis (AP), and CT Chest Abdomen Pelvis (CAP), as well as compare and discuss the derived DRLs with the most recently published DRLs in other countries.

## 2. Materials & Methods

### 2.1. Data Collection

In this retrospective study, dosimetric data were collected from CT paediatric patients’ scans available on the picture archiving and communication systems (PACS) at Sultan Qaboos University Hospital (SQUH; namely, Centre 1) and Royal Hospital (RH; namely, Centre 2). The radiation dose monitoring (RDM) and Al-Shifa hospital information system were used to extract the CT dosimetric data from Centre 1 and Centre 2, respectively. All patients’ scans were performed over the period from 2020 to 2024. Radiologists and medical physicists identified the four most common CT imaging examinations. These included CT Head, CT Chest, CT Abdomen Pelvis (AP), and CT Chest Abdomen Pelvis (CAP). Paediatric CT scans were all performed either on the Siemens SOMATOM Force 256 slice or Siemens SOMATOM Force 64 slice or Siemens SOMATOM Definition Flash (Siemens Healthineers, Erlangen, Germany). Dosimetric CT data were obtained from routine diagnostic scans performed on SOMATOM Force scanners at Centre 1, whereas Centre 2 operated both the Force 256 and the Definition Flash scanners. The use of CT dosimetric dose data acquired on different scanner models is an added value in terms of reliability and robustness in DRL studies and is expected to reflect local practice. All scanners were subject to strict quality assurance programs, including planned maintenance by the manufacturer, as well as daily, weekly, and annual quality control (QC) tests, along with post-repair QCs performed by local medical physicists. Additionally, despite both centres following quality control procedures, differences in the implemented scanning protocols such as scan length and automatic exposure control features may contribute to the variation in radiation dose across hospitals.

The total number of CT dosimetry records was collected from 5956 patient scans across both hospitals. The collected paediatric CT imaging was categorized into four paediatric age groups as recommended by ICRP 135 [12]: <1 year, 1–5 years, 6–10 years, and 11–15 years. Additionally, patients were classified into four weight groups according to the recommendations of “Radiation Protection N^o^ 185: European Guidelines on Diagnostic Reference Levels for Paediatric Imaging” [28]: <15 kg, 15–<30 kg, 30–50 kg, and >50 kg.

CT dosimetric data were collected from the dose reports generated by the CT acquisition and processing workstation at the completion of the patient study. To ensure the estimation of reliable and relevant DRLs, only diagnostic CT scans carried out using standard of care scanning protocols and approved by the quality management committee were considered in this study. This facilitates the comparison of the results with similar studies performed in other countries. A structured Excel form was developed and used by the researchers to collect data at both hospitals. CT dosimetry quantities (CTDI_vol_, DLP, exposure settings (kVp and mAs)) and patient demographics (age and weight) that were documented on the PACS or RIS radiology systems were collected from all patients’ scans.

### 2.2. Data Analysis

Microsoft Excel (Office 365), Minitab Statistical Software—Web App and OriginPro 2019 were used for data processing, statistical analysis, and charts generation presented in the ‘Results’ and ‘Discussion’ sections. Initially, Andreson–Darling normality tests were performed using Minitab to assess the distribution of continuous data. The normality tests identified that non-parametric tests were appropriate. Descriptive statistics, including minimum, median, maximum, interquartile ranges (IQRs), and 95% confidence intervals (CIs), for CTDIvol and DLP values across all CT imaging examinations were estimated. These median values were considered as local ‘typical values for DRLs’ and were subsequently compared to DRLs reported in recent studies. The Mann–Whitney U and Kruskal–Wallis tests were used to assess differences between the DRL values of the two centres and differences between age- and weight-based DRLs, respectively. These statistical indicators enhance transparency and allow a more robust comparison of dose distributions and paediatric scanning protocols between institutions. A significance level of 0.05 was applied for all statistical tests.

## 3. Results

### 3.1. Characteristics of Collected Data

Table 1 shows the CT acquisition parameters for the different imaging examinations performed at Centre 1 and Centre 2. Table 1 indicates that at Centre 1, head and chest examinations have the highest kVp values, while at Centre 2, all examinations fall within the same range of kVp. Generally, a kVp of 70 to 120 is predominantly used in CT imaging scans for both hospitals, with kVp 120 employed for overweight patients. Additionally, the mean mAs is higher for CAP in Centre 1, while head examinations exhibit the highest mean mAs in Centre 2.

### 3.2. Descriptive Statistics

Descriptive statistics, including sample sizes (N) for patient demographics and scan parameters across the two centres, are summarized in Table 2. Interquartile ranges (IQRs) and 95% CIs were used to describe data variability and estimate precision. The median age and weight were slightly higher in Centre 1, with broader IQRs suggesting a more diverse patient population. Notably, CTDI_vol_ and DLP values were consistently higher in Centre 1, accompanied by wider IQRs, indicating differences in scanning protocol acquisition parameters. In contrast, Centre 2 showed more consistent technical parameters, particularly in mAs and KVp, reflecting consistent use of scanning protocols. Overall, the CIs confirmed the statistical stability of the data, supporting their suitability for establishing local DRLs.

### 3.3. Age-Based Classification of DRLs

#### 3.3.1. Patients’ Demographics

A total of 5956 paediatric patient scans were recorded from the two centres based on their age in terms of CTDI_vol_ (mGy) and DLP (mGy.cm) values. As Table 3 shows, the percentages of collected data are 90% in Centre 1 and 10% in Centre 2, while the percentages per age group are 10.9% for <1 year, 30.8% for 1–5 years, 24.8% for 6–10 years, and 33.5% for 11–15 years. The elevated discrepancy in the amount of collected data between the two hospitals was due to the methods employed for data collection. At Centre 1, data were extracted from the RDM system and then automatically transferred to an Excel sheet, ensuring efficiency and consistency. In contrast, at Centre 2, data were manually retrieved from the Al-Shifa management and storage system, which is inherently more time-consuming and prone to human errors. Additionally, the highest frequency of CT examinations was observed in the older age group, while the lowest frequency was recorded in the youngest age group due to the limited number of referrals for this age category, especially for CAP scans.

#### 3.3.2. Estimation of DRLs

DRL values for all categories of CT imaging examinations were determined as the median of the CTDI_vol_ and DLP dose distributions. Table 4 summarizes the DRL values for all CT examinations across four age groups. It includes the median values for Centre 1, Centre 2, and the average across participating hospitals, as well as the ranges for CTDI_vol_ (mGy) and DLP (mGy.cm) values.

The median values for CTDI_vol_ and DLP exhibit a wide range, which reflects the variability in patients’ weight. Large differences in median CTDI_vol_ and DLP values were observed between examination types, with the largest disparity occurring in Head examinations and the smallest in Chest examinations for both CTDI_vol_ and DLP values.

The statistical analysis presented in Table 4 is discussed in detail in Section 4.1, ‘Age-based Classification of DRLs’, under the ‘Discussion’ Section.

### 3.4. Weight-Based DRLs Classification

#### 3.4.1. Patients’ Demographics

A total of 1446 paediatric patients’ doses were extracted from the two hospital sites based on their weight in terms of DLP (mGy.cm) and CTDI_vol_ (mGy) values. The percentages of collected data were 57.4% in Centre 1 and 42.6% in Centre 2. The percentages per weight group were as follows: 33.7% for <15 kg, 38.1% for 15− <30 kg, 15.3% for 30–50 kg, and 6% for >50 kg. The discrepancy in the volume of collected data was due, as stated in the Section “Age-based classification of DRLs”, to the automatic data collection at Centre 1 and manual data collection at Centre 2. The highest frequency of CT examinations was observed in the first weight group (<15 kg), while the lowest frequency was recorded in the fourth weight group (>50 kg). Table 5 presents the overall frequencies for all types of paediatric imaging examinations.

#### 3.4.2. Estimation of DRLs

From Table 6, median values for CTDI_vol_ and DLP exhibit a wide range, which reflects the variability in patient weight. Large differences in median CTDI_vol_ and DLP values were observed between examination types, with the largest disparity occurring in the head examination and the smallest in the chest examination.

The statistical analysis presented in Table 6 is discussed in detail in Section 4.2, ‘Weight-based Classification of DRLs’, subsection under the ‘Discussion’ Section.

## 4. Discussion

Unlike previous studies that investigated CT paediatric DRLs based either on age or weight, the present study categorised the collected data into four paediatric groups based on both age and weight. A comprehensive comparison of the DRLs between the two centres as well as with DRLs established in other countries is presented in the following sections.

### 4.1. Age-Based Classification of DRLs

#### 4.1.1. Comparison of Doses from Both Centres

The comparison of median CTDI_vol_ (mGy) and DLP (mGy·cm) values between Centre 1 and Centre 2 across different imaging protocols and age groups (Table 4) reveals notable dose differences. For the Head examination, Centre 1 consistently reports higher CTDI_vol_ values across all age groups, except for the 11–15 age group, whereas the CTDIvol at Centre 2 is only 32% of that at Centre 1. Similarly, DLP values are higher at Centre 1 for all age groups, with the most considerable difference seen in the 11–15 age group (219%; 537 mGy·cm vs. 2:245 mGy·cm). For the Chest examination, Centre 2 generally records higher CTDI_vol_ and DLP values compared to Centre 1, especially in younger age groups. For instance, in children under 1 year, Centre 1 has 50% and 40% lower CTDI_vol_ and DLP, respectively, compared to Centre 2. This trend continues for ages 1–10, where Centre 2 consistently shows higher values. However, for the 11–15 age group, both institutions report similar CTDI_vol_ values (3 mGy), with a DLP that is 15% lower at Centre 2.

The AP examination also demonstrates a trend where Centre 2 records slightly higher CTDI_vol_ and DLP values in younger age groups, but Centre 1 surpasses Centre 2 in older children. For instance, in children under 1 year, Centre 1 CTDI_vol_ and DLP are 30% and 32% lower, respectively, compared to Centre 2. However, in the 11–15 age group, Centre 1 reports CTDI_vol_ and DLP DRL values that are 36% and 27% higher, respectively, compared with Centre 2. Lastly, in the CAP examination, Centre 2 generally records higher values, particularly in the 1–5 and 6–10 age groups. For children aged 1–5, Centre 2 records CTDI_vol_ and DLP values that are 77% and 48% higher, respectively, than those of Centre 1. The difference continues in the 6–10 age group, where Centre 1 DLP is less than half that of Centre 2 (47%). However, for the 11–15 age group, both institutions report similar CTDI_vol_ values, though Centre 1 achieved a DLP that is 42% lower compared with Centre 2.

Overall, the inter-centre comparison for the four age categories identified the imaging examinations (Head examination at Centre 1 and Chest, AP and CAP examinations at Centre 2) that deserve higher attention in terms of radiation dose optimisation.

#### 4.1.2. CTDI_vol_ and DLP DRLs

As shown in Table 4, the Mann–Whitney test indicates that significant differences (*p* < 0.001) in median CTDI_vol_ and DLP values were observed across all age groups in head CT and chest CT examinations, with Centre 1 consistently showing higher median values. In contrast, there was no statistically significant differences in the older age groups for both centres, especially in median values of CTDI_vol_ and DLP for AP and CAP examinations. This reflects similarities between technical acquisition parameters and scanning practice (e.g., AP CTDI_vol_ median values for the 6–10 year age group with a *p* value of 0.805).

**Figure 1:**
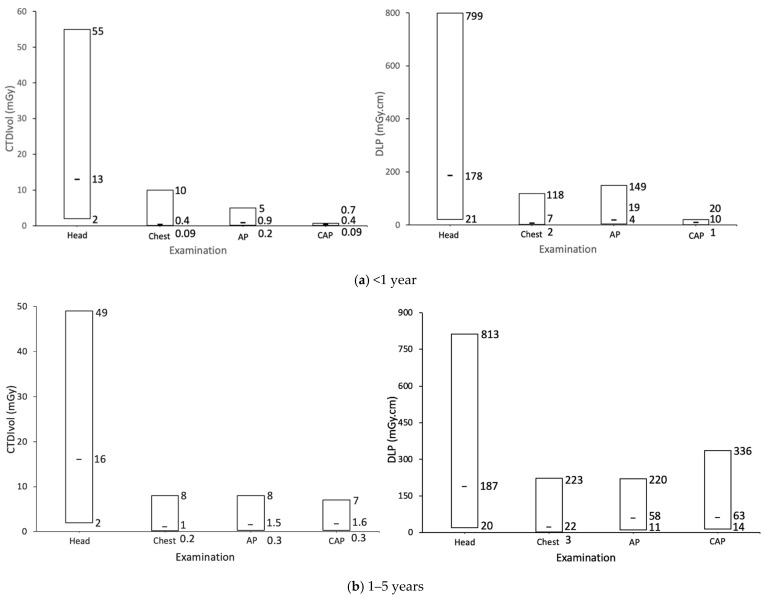

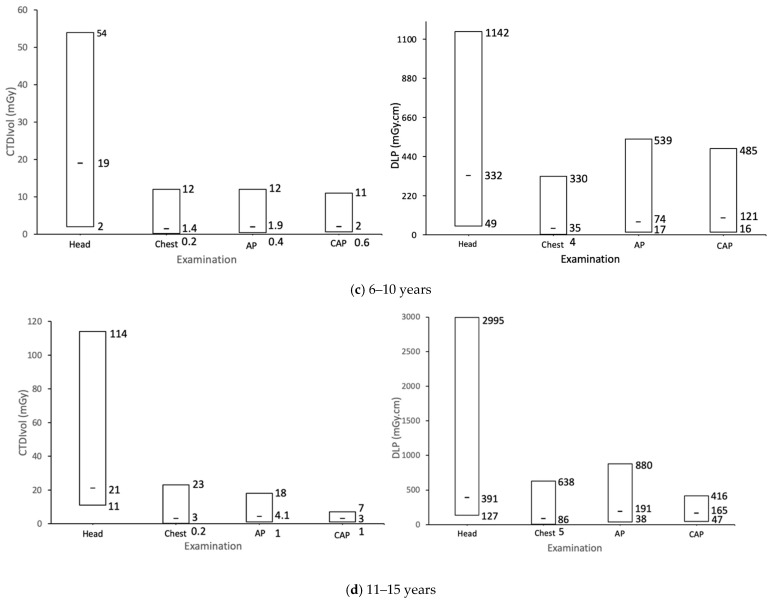
Bar charts of the minimum, median, maximum CTDI_vol_, and DLP for Head, Chest, AP, and CAP CT examinations for different age groups. Numeric values in each bar represent minimum (**bottom**), median (**middle**), and maximum (**top**) value.

Figure 1 illustrates the distribution of DRLs for paediatric CT examinations (Head, Chest, AP, and CAP) across different age groups (<1 year, 1–5 years, 6–10 years, and 11–15 years). The data reveal variations in dose levels depending on examination type, patient age, and examination adjustments aimed at balancing radiation exposure with diagnostic image quality. Head CT scans consistently exhibit the highest radiation doses across all age groups, which is expected due to the necessity for adequate X-ray penetration of the skull to produce diagnostic image quality. The distribution of Head CT doses is moderately right-skewed, with most values on the lower end and fewer high-dose cases, reflecting variation based on individual patient characteristics.

Chest and AP scans show a right-skewed distribution, where most examinations involve lower doses, but some cases require higher radiation levels due to extended scan duration and scan length defined by the operator. This variability indicates large variations in scanning protocols due to patient-specific factors (e.g., weight) and varying clinical requests (e.g., clinical indication). In contrast, CAP examinations exhibit a normal dose distribution across the youngest and oldest age groups. This demonstrates a consistent approach to CAP scanning protocol implementation in terms of dose exposure settings and selection of imaging range.

The rise in radiation dose with age across all scan types suggests that exposure settings are adjusted to accommodate patient size and maintain adequate image quality. The wide variation observed in Chest and AP scans highlights the necessity for improved scanning protocols to minimize unnecessary dose discrepancies, while the more consistent dose level in Head and CAP scans illustrates the benefits of using common local scanning practices. These findings emphasize the need for ongoing optimization of paediatric CT protocols to ensure effective dose management and minimize radiation risks without compromising diagnostic outcomes.

#### 4.1.3. Comparison to Other CT Paediatric DRLs

The comparison of the paediatric CT diagnostic reference levels (DRLs) from the present study with those reported in international studies reveals a consistent trend of lower radiation doses across most examination types and age groups, as shown in Table 7.

In this study, the CTDI_vol_ values for head examinations range from 13 to 21 mGy, noticeably lower than those reported in other countries: 23–69 mGy in Malaysia [29], 23–27 mGy in Morocco [30], 17–34 mGy in China [31], and 30–60 mGy in Japan [21]. The DLP values, ranging from 187 to 391 mGy·cm, are also considerably lower than the values reported in these studies, showing a reduction of 25–60%. This suggests effective local management of doses and optimization of protocols in head CT scanning. A similar pattern is found in chest CT examinations, where the CTDI_vol_ values in this study (0.4–3 mGy) are markedly lower than those reported in the United States (1.6–7.2 mGy) [32,33], Japan (3–6.5 mGy) [21], Malaysia (3.7–6.2 mGy) [29], and China (1.5–4.6 mGy) [31]. Additionally, the DLP values, spanning from 7 to 86 mGy·cm, are 70–90% lower than the abovementioned studies, indicating significant reductions in both dose per volume and scan length, especially among younger age groups who are more sensitive to radiation.

In AP CT scans, the CTDI_vol_ values found in this study (0.9–4.1 mGy) are notably lower than those from the US (2.4–7.9 mGy) [32] and Japan (5–9 mGy) [21]. They are instead comparable to results reported from Saudi Arabia [33] for the 1–5 year age group. However, the DLP values (19–191 mGy·cm) are 9–158% higher than the findings from the Saudi study, despite the lower CTDI_vol_. This difference implies that the lengths of scans used in AP examinations may exceed what is clinically necessary, a concern raised during recent local clinical governance meetings. In CAP CT scans, the CTDI_vol_ values in this study (0.4–3 mGy) are much lower than those found in the US (2.7–9.1 mGy) [32] and Malaysia (3–11.7 mGy) [29], reflecting dose reductions of up to 90%. The DLP values also align with this trend, showing a reduction of 60–90% across all age groups. Overall, these comparisons highlight effective dose optimization in the scanning protocols for head, chest, and CAP examinations, consistent with radiation protection principles in paediatric imaging. The consistently lower CTDI_vol_ and DLP values indicate that radiographers are successfully implementing and running specialized paediatric protocols, particularly in sensitive areas such as the brain and chest.

### 4.2. Weight-Based Classification of DRLs

#### 4.2.1. CTDI_vol_ and DLP DRLs

As shown in Table 6, when stratified by weight, statistically significant differences in median CTDI_vol_ and DLP values were found in most weight groups between the two centres, particularly for head and chest CT examinations in paediatric patients under 50 kg (*p* < 0.001). Centre 1 consistently demonstrated higher median CTDI_vol_ and DLP values in head CT, while Centre 2 reported higher median values for CTDI_vol_ and DLP in chest and CAP examinations, especially in lighter patients. In contrast, no significant differences in median CTDI_vol_ and DLP values were found for chest and CAP examinations for patients over 50 kg (*p* = 0.580 and *p* = 0.134, respectively). Overall, these findings reflect more aligned practices in terms of scanning protocol parameters (exposure parameters and the selected imaging range) in the two centres in heavier paediatric patients.

**Figure 2:**
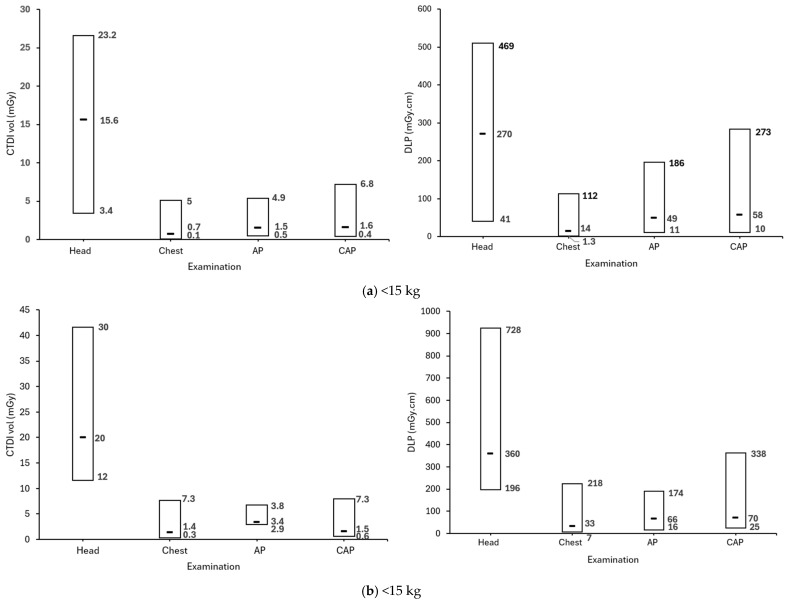

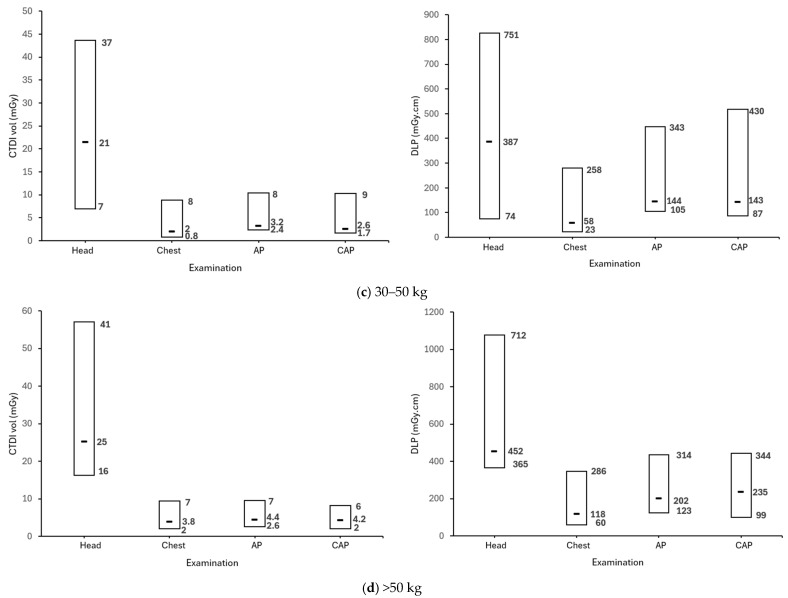
Bar charts of the minimum, median, maximum CTDI_vol_, and DLP for Head, Chest, AP, and CAP CT examinations for the different weight groups. Numeric values in each bar represent minimum (**bottom**), median (**middle**), and maximum (**top**) value.

Figure 2 illustrates the distribution of DRLs for paediatric CT examinations (head, chest, AP, and CAP) across the different weight groups (<15 kg, 15−<30 kg, 30–50 kg and >50 kg). The median CTDI_vol_ (mGy) range was found to be 3.4–40.9 for the head, 0.1–7.4 for the chest, 0.5–6.9 for the AP, and 0.4–6.2 for the CAP. The DLP (mGy.cm) was found to be 712 for the head, 13–286 for the chest, 11–314 for AP and 10–344 for the CAP. The lowest CTDI_vol_ and DLP values were observed in the lightest group of <15 kg. The values of CTDI_vol_ and DLP increased with weight, reaching the highest values for the heaviest group of >50 kg. These findings are in agreement with previous studies that have demonstrated a relationship between median values of CTDI_vol_ and DLP with weight [21,34,35,36,37]. As patient weight increases, higher radiation output is typically needed to ensure adequate image quality, which leads to an increase in both CTDI_vol_ and DLP values due to greater body size and scan length. Head CT scans consistently exhibit the highest radiation doses across all weight groups, which is expected, as generating diagnostic quality images of the brain requires more energy to pass through the dense structure of the skull. The CTDI_vol_ distribution for head CT shows a clear right-skewed pattern. For chest, AP, and CAP scans a right-skewed distribution is observed as well, indicating these findings of DRLs do not mean to differentiate between good or bad clinical practice. The use of DRLs for paediatric patients is likely to raise radiation exposure awareness among medical staff in radiology departments, encouraging safer CT paediatric practices and aiding in acquiring an appropriate diagnostic CT image quality required for paediatric examinations.

#### 4.2.2. Comparison to Other CT Paediatric DRLs

As there are limited data available from Oman, we benchmarked against international data, as shown in Table 8. The DRL values for CT examination of chest, pelvis, and chest and pelvis were lower than those reported in Japan [21,34], Germany [35], Saudi Arabia [33], Greece [36] and Europe [37]. The head scan results were not compared with other published reports since no data were reported in these studies. For chest scans, the values of CTDI_vol_ observed in this study for most weight groups were lower compared to data from other countries except Greece [36], where the values were comparable in the heaviest groups of 30–50 kg and <50 kg. Our DLP values are less than those in other countries, indicating that lower scan lengths were present in our data. For AP and CAP scans, the values of CTDI_vol_ and DLP values observed in this study for most weight groups were generally 40–65% lower when compared to data from other countries, but not those for AP and CAP scans in Saudi Arabia [33], where the values were comparable for all weight groups except in the lightest group of <15 kg.

These differences may be attributed to several factors. One major reason is the variation in scanner type and technology; some centres use newer CT systems that are more efficient in reducing doses. Another important factor is the scanning protocol itself, including settings like tube current, voltage, scan length, and whether dose-saving features like automatic exposure control are used properly. Also, the level of experience among radiologists and how well protocols are followed can affect dose values.

The Kruskal–Wallis test indicates a statistically significant difference in CTDI_vol_ and DLP DRLs between age- and weight-based groups across the different examinations, with a *p*-value of 0.006. Nonetheless, the age− and weight-based DRLs derived in the present study are in good agreement with several recently published paediatric CT DRLs.

From a practical implementation perspective, the findings of this study suggest that age-based grouping of paediatric subjects may allow for more consistent benchmarking and easier integration into clinical practice. A major challenging factor in paediatric imaging is that age is more frequently and accurately documented in medical records than weight, making it a more practical and reliable parameter for establishing and implementing DRLs. Therefore, age-based DRLs appear to be more effective than weight-based DRLs for dose optimisation in paediatric CT imaging. Nonetheless, weight-based DRLs could offer more tailored adjustment of exposure parameters, potentially leading to lower doses for individual patients.

The study has several limitations to note. First, manual collection of CT dose data was time-consuming, especially compared to automated methods using dose management software [34,36,37]. Second, some patients’ weight data were missing, which led to the exclusion of their scans. Third, the limited frequency of some examinations could extend significantly the data collection period, highlighting the need for team-based efforts. Fourth, the data were collected from only two large hospitals. Including more hospitals from across the country in future studies would help establish more reliable and robust national DRLs that better reflect national imaging practices. Finally, collecting more paediatric CT dose data may provide a clearer understanding of current clinical approaches [34,35]. Additionally, the optimisation of radiation doses through DRLs becomes more meaningful if radiation dose is evaluated alongside the desired image quality required for diagnosis [19,34]. In this context, Rehani et al. [13] introduced the concept of acceptable quality dose. This concept aligns with the diagnostic imaging vetting process and supports the development of more robust and clinically relevant DRLs that enhance optimisation efforts in routine radiology practice.

## 5. Conclusions

In the present study, age- and weight-based DRLs for the four most common paediatric CT examinations were established for dosimetric data collected from the two largest tertiary hospitals in Oman. The CTDI_vol_ and DLP DRLs reveal noticeable differences in dose distribution between the two hospitals, highlighting the need for review and optimisation of some scanning protocols, especially for Chest and AP examinations. Additionally, the estimated DRLs compare well with those published in other countries, assuring a good adherence to radiation protection principles and international safety standards. To obtain more representative and reliable dose distributions, the derived DRLs should be updated by including paediatric CT data from a broader range of hospitals across the country. This study also underscores the importance of using radiation dose monitoring tools to streamline and accelerate the DRL estimation process. Based on this first study of paediatric CT DRLs in Oman, we recommend developing national DRL guidelines, implementing dose-monitoring software in clinical practice, and providing regular training for radiology staff to support dose optimization and patient safety.

## Figures and Tables

**Table 1:** Patients’ weight and acquisition parameters in CT paediatric examinations at Centre 1 and Centre 2.

Examination	Weight (Range, kg)	kVp	mAs	Pitch Factor		Rotation Time (s)
Centre 1						
Head	0.45–80	70–150	18–693	0.35–1.20		0.50–1.00
Chest	1–105.5	70–150	17.5–693	0.55–3.00		0.25–1.00
AP	1.65–81	70–120	21–693	0.60–1.90		0.25–1.00
CAP	1.55–108	70–120	28–693	0.60–1.90		0.25–0.50
Centre 2						
Head	1.74–69.50	70–120	69–400		0.8–0.55	0.50–1.00
Chest	2.33–69.50	70–120	12–275		1.9–3.2	0.25–0.28
AP	2.33–69.50	70–120	26–200		1.9–2.5	0.25–0.28
CAP	6.50–69.50	70–120	44–200		1.9–2.5	0.25–0.28

**Table 2:** Descriptive statistics of CTDI_vol_ and DLP across Centre 1 and Centre 2, including medians, IQRs, and 95% confidence intervals (CI).

Variable	Centre	N	Mean	StDev	Median	IQR	95% CI
Age (year)	Centre 1	5341	7.57	5.18	7	9.99	(7–7)
	Centre 2	616	5.89	4.03	6	8	(7–7)
Weight (kg)	Centre 1	830	21.80	16.29	18	17.68	(16.6–18)
	Centre 2	616	20.01	12.83	17.35	15.41	(16.6–18)
CTDI_vol_ (mGy)	Centre 1	5341	12.81	11.53	11.05	19.09	(10.28–11.23)
	Centre 2	616	8.63	7.25	6.495	12.69	(10.28–11.23)
DLP (mGy.cm)	Centre 1	5341	254.50	211.03	216.89	332.29	(200.95–216.89)
	Centre 2	616	170.14	124.03	146.5	207.63	(201–216.89)
KVp	Centre 1	5341	77.25	95.06	32.75	139.75	(46.9–54.5)
	Centre 2	616	91.66	13.05	100	20	(46.94–54.46)
mAs	Centre 1	5341	77.25	95.06	32.75	139.75	(46–53)
	Centre 2	616	139.33	56.76	133	78.75	(46–53)

**Table 3:** Volume of CT scans performed across different age groups, hospitals, and examinations.

	<1 Year	1–5 Years	6–10 Years	11–15 Years	Total
Centre					
Centre 1	573	1616	1275	1876	5340
Centre 2	74	219	201	122	616
All	647	1835	1476	1998	5956
Examination					
Brain	409	1090	774	820	3093
Chest	182	496	351	532	1561
AP	562	179	272	638	1651
CAP	15	73	78	60	226

**Table 4:** The estimated median values for CTDI_vol_ and DLP for Centre 1 and Centre 2 combined, according to the four age groups.

Imaging Protocol		CTDI_vol_ (mGy)					DLP (mGy.cm)				
	Age Group (Year)	Range	Median Centre 1	Median Centre 2	Median	*p*-Value	Range	Median Centre 1	Median Centre 2	Median	*p*-Value
Head	<1	2–55	15	11	13	<0.001	21–799	228	145	187	<0.001
	1–5	2–49	18	14	16	<0.001	20–813	316	236	276	<0.001
	6–10	2–54	20	17	19	<0.001	49–1142	359	304	332	<0.001
	11–15	11–114	31	10	21	<0.001	135–2995	537	245	391	<0.001
Chest	<1	0.1–10	0.3	0.6	0.4	0.006	2–118	4	10	7	0.006
	1–5	0.2–8	0.5	1.4	1	<0.001	3–223	10	33	22	<0.001
	6–10	0.2–12	0.7	2	1.4	<0.001	4–330	15	54	35	<0.001
	11–15	0.2–23	3	3	3	0.471	5–628	93	79	86	0.411
AP	<1	0.2–5	0.7	1	0.9	0.033	21–149	15	22	19	0.054
	1–5	0.3–8	1	2	1.5	0.067	11–220	42	73	58	0.018
	6–10	0.4–12	1.5	2.3	1.9	0.805	17–539	60	87	74	0.960
	11–15	1–18	5	3.2	4.1	0.043	38–880	220	161	191	-
CAP	<1	0.1–0.7	0.4	-	0.4	-	1–20	10	-	10	-
	1–5	0.3–7	1	2.2	1.6	0.184	14–336	41	84	63	0.012
	6–10	0.6–11	2	2	2	0.726	16–485	114	128	121	0.677
	11–15	1–7	3	3	3	0.967	47–416	122	207	165	0.185

**Table 5:** Volume of CT scans performed across different weight groups, centres, and examinations.

	<15 kg	15−<30 kg	30–50 kg	>50 kg	Total
Hospital					
Centre 1	340	291	143	56	830
Centre 2	247	260	78	31	616
All	487	551	221	87	1446
Examination					
Brain	253	204	81	23	561
Chest	193	138	42	20	393
AP	89	137	64	37	327
CAP	52	72	34	20	178

**Table 6:** The estimated median values for CTDI_vol_ and DLP for Centres 1 and 2 combined according to the four weight groups.

Imaging Protocol		CTDI_vol_ (mGy)					DLP (mGy.cm)				
	Weight Group (kg)	Range	Median Centre 1	Median Centre 2	Median	*p*-Value	Range	Median Centre 1	Median Centre 2	Median	*p*-Value
Head	<15	3.4–23.2	19.2	12	15.6	<0.001	41–469	339	202	270	<0.001
	15−>30	11.6–30.1	23.3	16.7	18.0	<0.001	196–728	420	300	360	<0.001
	30–50	7–36.6	24.9	17.8	21.4	<0.001	74–751	448	325	387	<0.001
	>50	16.2–40.9	30.1	20.5	25.3	0.009	365–712	538	365	452	0.001
Chest	<15	0.1–5	0.4	1.1	0.7	<0.001	13–112	6.9	21	14	<0.001
	15−>30	0.3–7.3	0.6	2.2	1.4	<0.001	7–218	13	53.8	33	<0.001
	30–50	0.8–8.1	1.0	3	2	0.008	23–258	31	85.7	58	0.008
	>50	2–7.4	4.1	3.5	3.8	0.580	60–286	129	108	118	0.175
AP	<15	0.5–4.9	0.9	2.1	1.5	<0.001	11–186	25	72	49	<0.001
	15−>30	2.9–3.8	1.4	2.2	3.4	0.017	16–174	49	83	66	0.024
	30–50	2.4–8.0	2.7	3.7	3.2	0.025	105–343	113	175	144	0.009
	>50	2.6–6.9	5.3	3.5	4.4	-	123–314	231	172	202	-
CAP	<15	0.4–6.8	1	2.2	1.6	0.005	10–273	31	84	58	0.002
	15−>30	0.6–7.3	1.4	1.7	1.5	0.849	25–338	61	78	70	1.000
	30–50	1.7–8.5	2.2	2.9	2.6	0.086	88–430	79	207	143	0.018
	>50	2.0–6.2	4	4.4	4.2	0.428	99–344	185	286	235	0.134

**Table 7:** DRL values in terms of CTDI_vol_ (mGy) and the DLP (mGy·cm) estimated in the present study compared with data reported in studies from Malaysia, Morocco, China, US, Japan, and Saudi Arabia (KSA).

Age Group (Year)	<1		1–5		6–10		11–15	
Quantity	CTDI_vol_	DLP	CTDI_vol_	DLP	CTDI_vol_	DLP	CTDI_vol_	DLP
Examination	Head							
Present study	13	187	16	276	19	332	21	391
Japan [21]	30	480	40	660	55	850	60	1000
Malaysia [29]	23	250	29	449	32	459	69	811
Morocco [30]	23	380	25	454	26	498	27	523
China [31]	17	260	24	340	27	434	34	434
Examination	Chest							
Present study	0.4	7	1	22	1.4	35	3	86
US [32]	1.6	31	2.4	58	2.9	95	7.2	272
Japan [21]	3	70	4	95	6.5	175	6.5	30
Malaysia [29]	3.7	47	4.2	67	6.2	126	6.2	156
China [31]	1.5	29	1.6	35	2.2	60	4.6	153
Examination	AP							
Present study	0.9	19	1.6	58	1.9	74	4.1	191
US [32]	2.4	60	2.9	100	4.6	170	7.9	358
Japan [21]	5	110	6	190	7.5	265	9	450
KSA [33]	-	-	1.5	29	2.4	66	5.7	74
Examination	CAP							
Present study	0.4	10	1.6	63	2	121	3	165
US [32]	2.7	89	3	109	4.3	204	9.1	437
Malaysia [29]	3	98	4.6	150	7.1	293	11.7	645

**Table 8:** DRL values in terms of CTDI_vol_ (mGy) and DLP (mGy·cm) estimated in the present study compared with data reported in previous studies from Greece, Japan, Germany, Europe, and Saudi Arabia.

Weight Group (Kg)	<15		15−<30		30–50		>50	
Quantity	CTDI_vol_	DLP	CTDI_vol_	DLP	CTDI_vol_	DLP	CTDI_vol_	DLP
Examination	Chest							
Present study	0.7	14.1	1.4	33.4	2	58.4	3.8	118.2
Saudi Arabia [33]	2.7	38.2	3.4	71.4	4.7	107.2	6.5	177
Greece [36]	2	42	2	61	2	61	3	110
Europe [37]	1	35	2	50	3	70	4	115
Japan [21]	2.5	38	4.5	61	5.5	155	6.5	225
Germany [35]	-	-	2.7	50	-	-	-	-
Examination	AP							
Present study	1.5	49	3.4	66	3.2	144	4.4	202
Saudi Arabia [33]	2.4	71.3	4.1	156.1	5.1	181.2	4.2	206
Greece [36]	-	-	2	85	2	149	3	226
Europe [37]	-	-	4	120	5	150	7	210
Japan [21]	2.5	65	6	165	6.5	305	8	360
Germany [35]	-	-	3	102	-	-	-	-
Examination	CAP							
Present study	1.6	58	1.5	70	2.6	143	4.2	235
Saudi Arabia [33]	2.6	61.2	2.9	80.5	2.9	129.3	4.7	230

## Data Availability

The data collected from the radiology storage systems at Sultan Qaboos University Hospital and Royal Hospital are only available for access by the local researchers. The data analysed or generated during the study are included in the manuscript.
